# Information Flow Pattern in Early Mild Cognitive Impairment Patients

**DOI:** 10.3389/fneur.2021.706631

**Published:** 2021-11-11

**Authors:** Haijuan He, Shuang Ding, Chunhui Jiang, Yuanyuan Wang, Qiaoya Luo, Yunling Wang, Alzheimer's Disease Neuroimaging Initiative

**Affiliations:** Department of Radiology, The First Affiliated Hospital, Xinjiang Medical University, Xinjiang, China

**Keywords:** resting state functional MRI, information flow, support vector classification, support vector regression, early mild cognitive impairment

## Abstract

**Purpose:** To investigate the brain information flow pattern in patients with early mild cognitive impairment (EMCI) and explore its potential ability of differentiation and prediction for EMCI.

**Methods:** In this study, 49 patients with EMCI and 40 age- and sex-matched healthy controls (HCs) with available resting-state functional MRI images and neurological measures [including the neuropsychological evaluation and cerebrospinal fluid (CSF) biomarkers] were included from the Alzheimer's Disease Neuroimaging Initiative. Functional MRI measures including preferred information flow direction between brain regions and preferred information flow index of each brain region parcellated by the Atlas of Intrinsic Connectivity of Homotopic Areas (AICHA) were calculated by using non-parametric multiplicative regression-Granger causality analysis (NPMR-GCA). Edge- and node-wise Student's *t*-test was conducted for between-group comparison. Support vector classification was performed to differentiate EMCI from HC. The least absolute shrinkage and selection operator (lasso) regression were used to evaluate the predictive ability of information flow measures for the neurological state.

**Results:** Compared to HC, disturbed preferred information flow directions between brain regions involving default mode network (DMN), executive control network (ECN), somatomotor network (SMN), and visual network (VN) were observed in patients with EMCI. An altered preferred information flow index in several brain regions (including the thalamus, posterior cingulate, and precentral gyrus) was also observed. Classification accuracy of 80% for differentiating patients with EMCI from HC was achieved by using the preferred information flow directions. The preferred information flow directions have a good ability to predict memory and executive function, level of amyloid β, tau protein, and phosphorylated tau protein with the high Pearson's correlation coefficients (*r* > 0.7) between predictive and actual neurological measures.

**Conclusion:** Patients with EMCI were presented with a disturbed brain information flow pattern, which could help clinicians to identify patients with EMCI and assess their neurological state.

## Introduction

Early mild cognitive impairment (EMCI) has been considered as the mildest neuropsychological impairment (including memory and cognitive deficit) state preceding Alzheimer's disease (AD) (1). The clinical manifestations of EMCI include mild loss of motor functions, speech difficulties, memory concerns, and decreased ability to read and write, which could be observed in the normal elderly population as well, making it difficult for clinical diagnosis (2–5). Cognitive assessments, serologic tests, cerebrospinal fluid (CSF) biomarkers, and genotypes contribute to early identification of EMCI and assessment of neurological state (6–9). However, cognitive assessments were time-consuming. Serologic tests and CSF examination were invasive and not available for all the potential patients with EMCI in clinical practice (10). Therefore, noninvasive objective biomarkers were warranted to accurately differentiate EMCI from normal elders and assess the neurological state (e.g., cognitive state and CSF biomarker levels).

Resting-state functional MRI (rs-fMRI) was first described by Biswal et al. in (11). Since then, it has been widely applied in healthy populations and patients with various neurologic, neurosurgical, and psychiatric disorders. Compared to task-based fMRI, rs-fMRI does not require subjects to perform any specific task and could reflect intrinsic relationships between the brain regions or brain networks in greater detail in neurodegenerative disorders (12). In fact, the task-based fMRI was more targeted to relationships between the brain functional areas and specific cognitive tasks (e.g., dorsal and ventral attention network activation in a short-term memory task, occipital and frontal gyrus activation in visuospatial memory task) (13, 14), while the low-frequency oscillations of the rs-fMRI signal were more associated with the spontaneous neural activity and can be used to depict the underlying intrinsic whole-brain functional connectivity, which accounts for various cognitive information processing in neurodegenerative disorders (12). rs-fMRI has been widely applied for non-invasively detecting brain functional alterations associated with the underlying pathogenies (e.g., amyloid aggregates) and cognitive decline in patients with MCI and AD (10, 15, 16). Accumulated evidence demonstrated that rs-fMRI could characterize the underlying functional alterations preceding observed structural changes in the early stage of AD (1, 10). Voxel- or region-based functional connectivity analyses have been proposed to disclose the underlying functional patterns in MCI, which depicts the information flow across spatially separated brain areas. Both the functional deficits and compensations were reported in patients with MCI, indicating a complex underlying mechanism in MCI (17). Even though the underlying information flow patterns regarding the hippocampus, prefrontal, and temporal cortex could identify EMCI from later MCI and AD (1, 18–20), the underlying brain information flow pattern in patients with EMCI was still undetermined.

Evidence demonstrated that effective connectivity (directed connectivity) characterizing the information flow patterns in MCI and AD was superior to conventional functional connectivity (nondirected connectivity) by using correlation-based methods (e.g., Pearson's correlation, partial correlation, coherence analysis). Granger causality analysis (GCA), especially linear GCA, has been widely applied to investigate underlying directed information flow in MCI and AD (15, 21). However, it was argued that the functional interactions between brain areas were not linear and might be misinterpreted by linear regression (22–24). Non-parametric multiplicative regression-GCA (NPMR-GCA) was a non-parametric method to reflect the non-linear interaction of signals by presenting the interaction in high-dimensional embedded linear space, which seemed superior to conventional GCA in interpreting the non-linear functional interaction of brain areas (25, 26).

Therefore, in this study, we aimed to investigate the brain information flow pattern in patients with EMCI by using NPMR-GCA and explored its clinical significance including differential diagnosis and neurological state assessment.

## Methods

### Alzheimer's Disease Neuroimaging Initiative (ADNI) Data Acquisition

The MRI images in this study were obtained from the ADNI database (http://adni.loni.usc.edu/, data in work were acquired from ADNI-1, ADNI-GO, and ADNI-2). The ADNI was launched in 2004 funded by the National Institute on Aging (NIA), the National Institute of Biomedical Imaging and Bioengineering (NIBIB), and supported by many pharmaceutical companies and foundations. The primary goal was to investigate the progression of early AD and MCI by various measurements including neuropsychological assessments, MRI and PET imaging, and other biological markers (e.g., CSF biomarkers) (27, 28).

High-resolution three-dimensional (3D) T1 and rs-fMRI images of 49 patients with EMCI and 40 age- and sex-matched healthy controls (HCs) were included in this study. The inclusion criteria of HC were as follows: (1) the Mini-Mental State Examination (MMSE) scores between 24 and 30; (2) the Clinical Dementia Rating (CDR) of 0; and (3) no other neurological or psychiatric disorders. The inclusion criteria of EMCI were as follows: (1) the MMSE scores between 24 and 30; (2) having memory complaint and objective memory loss measured by education adjusted scores on the Wechsler Memory Scale-Revised Logical Memory II Story A score (a maximum score of 25): EMCI was assigned for a score of 9–11 for 16 or more years of education, a score of 5–9 for 8–15 years of education, or a score of 3–6 for 0–7 years of education; (3) the CDR of 0.5; (4) preserved activities of daily living; (5) no significant impairment in other cognitive domains; and (6) no dementia.

All the MRI images were acquired on a 3T Philips MR scanner. The MR protocol parameters of 3D T1 and rs-fMRI were as follows. 3D T1: 3D sagittal acquisition with magnetization-prepared rapid gradient-echo (MP-RGAE), repetition time (TR)/echo time (TE) = 6,700 ms/3.1 ms, flip angle (FA) = 9°, spatial resolution = 1 × 1 × 1.2 mm, matrix size = 256 × 256, and slice number = 170 and rs-fMRI: multislice axial acquisition with gradient echo-echo planar imaging (GRE-EPI), TR/TE = 3,000 ms/30 ms, FA = 80°, in-plane resolution = 3 mm × 3 mm, slice thickness = 3.3 mm, matrix size = 64 × 64, slice number = 46, and dynamics = 140.

Clinical measures including neuropsychological evaluation [ADNI-composite assessment of memory (ADNI-MEM) and ADNI-executive function (ADNI-EF) and CSF biomarkers [the accumulation of amyloid β (Aβ), tau protein, and phosphorylated tau (pTau) protein] were also obtained in a subset and used in this work to reflect the neurological state (Table 1).

**Table 1:** Demographics and clinical measures of the HC and EMCI subjects.

	**HC** **(***n*** = 40)**	**EMCI** **(***n*** = 49)**	***P*** **value**
Age (mean±SD, year)	75.1 ± 6.31	72.2 ± 6.72	0.34^#^
Female/Male	22/18	25/24	0.71^*^
ADNI-MEM (mean±SD)	1.0 ± 0.55 (n=36)	0.5 ± 0.57 (*n =* 44)	<0.001^#^
ADNI-EF (mean±SD)	0.8 ± 0.75 (*n =* 36)	0.5 ± 0.81 (*n =* 44)	0.053^#^
Aβ (mean±SD, pg/ml)	188.5 ± 48.79 (*n =* 29)	185.9 ± 62.26 (*n =* 37)	0.85^#^
Tau (mean±SD, pg/ml)	73.4 ± 35.43 (*n =* 29)	93.1 ± 64.66 (*n =* 37)	0.12^#^
pTau (mean±SD, pg/ml)	36.1 ± 17.69 (*n =* 29)	42.3 ± 26.25 (*n =* 37)	0.26^#^

*HC, healthy controls; EMCI, early mild cognitive impairment; Aβ, amyloid β; ADNI-MEM, Alzheimer's Disease Neuroimaging Initiative-composite assessment of memory ADNI-EF, ADNI-executive function; pTau, phosphorylated tau*.

* *Chi-squared test, p < 0.05 deemed as statistically significant*.

# *Two sample Student's t-test, p < 0.05 deemed as statistically significant*.

### MRI Processing

**Figure 1:**
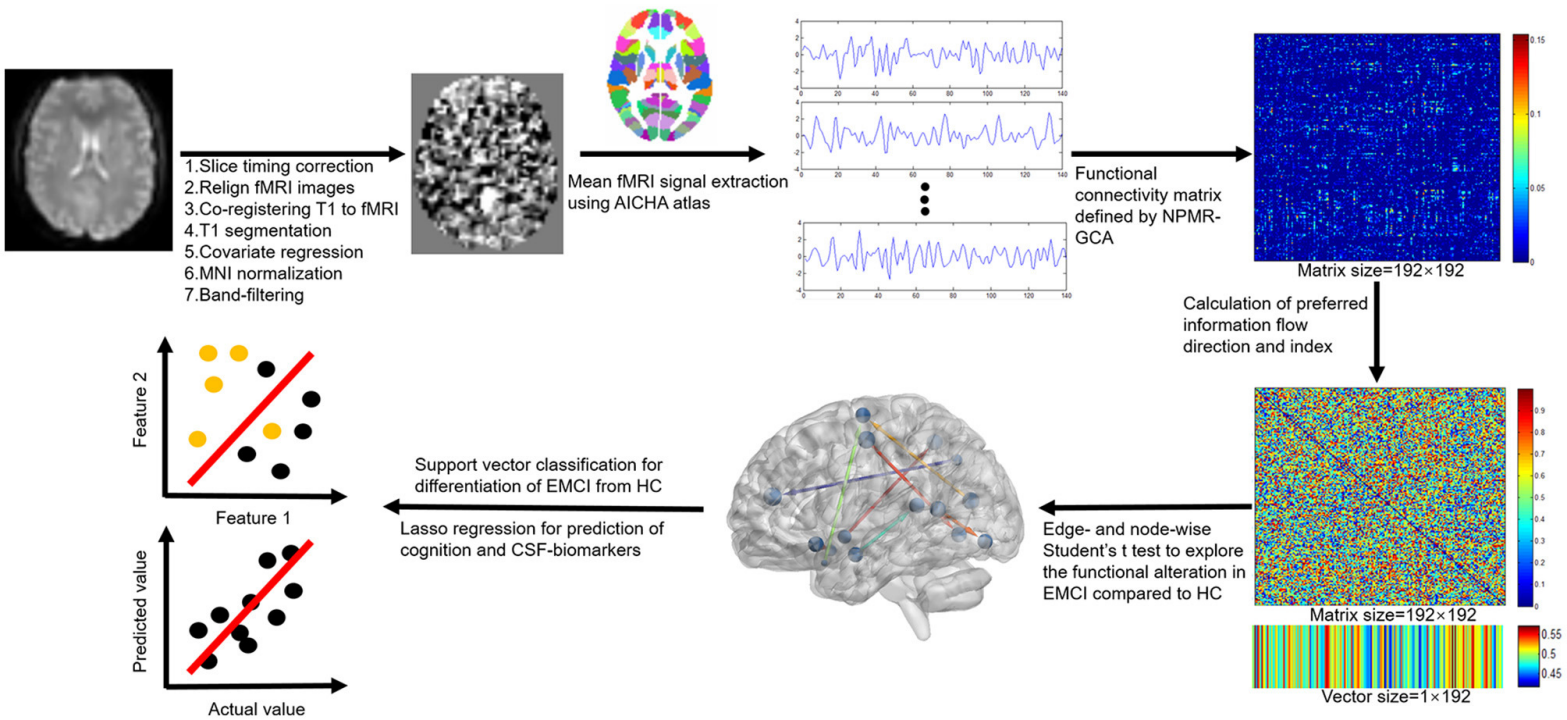
Functional MRI (fMRI) processing and analysis flowchart of the study.

Resting-state functional MRI images were preprocessed by using the Data Processing & Analysis for Brain Imaging (DPABI, Beijing, China) (version 4.4, http://rfmri.org/dpabi) software. Main processing procedures (Figure 1) included: (1) slice timing correction to correct for slice-dependent delays achieved by shifting the time series of each slice to temporally align all the slices to a reference time point (middle slice); (2) head motion correction by realigning the fMRI volumes to the mean volume; (3) coregistering T1 image to mean fMRI volume to obtain the forward and backward transformation matrices; (4) segmentation of T1 image to obtain the gray matter, white matter, CSF, and the normalization matrix (forward transformation matrix) of T1 to the Montreal Neurological Institute (MNI) space; (5) regression of covariates to remove the potential effects of linear trend, head motion (24 head motion parameters), global signal, and signals within white matter and CSF; (6) warping the fMRI images into the MNI space by the previous backward transformation matrix of coregistering T1 to fMRI and forward transformation matrix of normalizing T1 to MNI space; and (7) band filtering the fMRI signals with a frequency range of 0.01–0.1 Hz to keep only the interesting frequencies and discard potential noise sources (noise or physiological signal). No image smoothing was performed to preserve the details of fMRI signals.

### Calculation of Preferred Information Flow Measures

Information flow measure was calculated by NPMR-GCA with home-developed MATLAB scripts (Matlab, 2019b, MathWorks, USA; see Supplemental Material). The functional Atlas of Intrinsic Connectivity of Homotopic Areas (AICHA) was adopted in this study (29) to define the spatially separated gray matter regions. This atlas has 192 separated brain labels (brain nodes) and each label incorporates the bilateral homotopic brain areas, which are deemed to have the same function.

**Figure 2:**
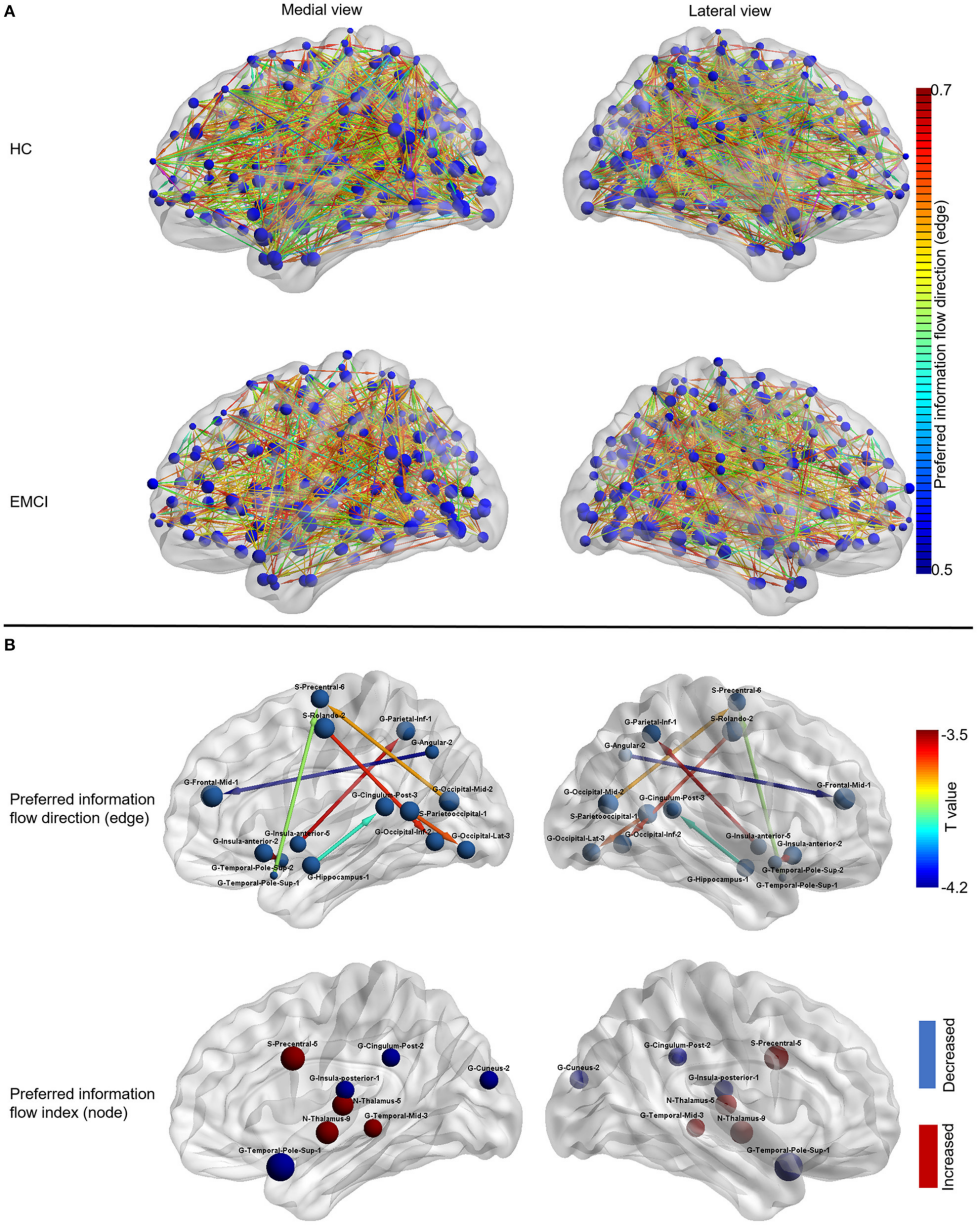
The information flow patterns in HC and EMCI. **(A)** The preferred information flow direction and preferred information flow index in HC and EMCI. The node size indicated the value of the preferred information flow index. The color of the directed edge indicated the value of preferred information flow direction (only directed edges with values > 0.5 were presented); **(B)** The alterations of information flow patterns in EMCI compared to HC. The first row presented the edge-wise statistical results of preferred information flow direction, only decreased preferred information flow direction in EMCI was displayed as the opposite preferred information flow direction was increased, which implies the identical information flow changes between the brain nodes; the Second row presented the node-wise statistical results of preferred information flow index, red nodes indicated the increased preferred information flow index and the blue nodes indicated the decreased preferred information flow index. HC, healthy controls; EMCI, early mild cognitive impairment; G, gyrus; S, sulcus.

Steps of information flow measure calculation were as follows:

Step 1: The mean fMRI signals were extracted for the 192 brain regions.
Step 2: The effective connectivity matrix between brain regions was constructed by NPMR-GCA. The functional connection was weighted by NPMR-GCA values presented by *GCA*(*i,j*) to characterize the directed information flow from brain region *i* to brain region *j*.
Step 3: The preferred information flow direction was defined as the relative information flow between each paired brain region by using *preferred*_*GCA* (*i,j*) = *GCA*(*i,j*)/[*GCA*(*i,j*)+*GCA*(*j, i*)] (Figures 1, 2A). A value of *preferred*_*GCA*(*i,j*)>0.5 indicated preferred information outputting of brain region *i* compared to brain region *j*, and a value of *preferred*_*GCA*(*i,j*) < 0.5 indicated preferred information receiving of brain region *i* compared to brain region *j*. A value of *preferred*_*GCA*(*i,j*) = 0.5 indicated no preferred information flow direction between brain regions *i* and *j*.
Step 4: Additionally, we defined the preferred information flow index [*preferred*_*GCA*(*i*)] of a brain region by averaging the preferred outputting direction across all the other brain regions (Figures 1, 2A), a *preferred*_*GCA*(*i*)>0.5 indicated preferred information outputting ability of a brain region at the whole brain level, and a *preferred*_*GCA*(*i*) < 0.5 indicated preferred information receiving ability.

### Statistical Analyses

Statistical analyses were conducted by using the Statistical Package for the Social Sciences (SPSS) (version 22.0, IBM Corporation, Armonk, New York, USA) and MATLAB scripts (Matlab, 2019b, MathWorks, USA). Categorical variables (e.g., sex) were displayed with percentages and analyzed by the chi-squared test. Continuous variable (e.g., age, neurological measures) was displayed with mean and SD. Data normality was analyzed by the Kolmogorov–Smirnov test. The Student's *t*-test was used for between-group comparison (EMCI vs. HC) if data were normally distributed; otherwise, the Mann–Whitney *U* test was used. A two-sided *p* < 0.05 was deemed as statistically significant.

The edge-wise Student's *t*-test was used to compare the preferred information flow direction between groups (EMCI vs. HC) and a two-sided false discovery rate (FDR)-corrected *p* < 0.05 was deemed as statistically significant. The node-wise Student's *t*-test was used to compare the preferred information flow index between groups and a two-sided FDR-corrected *p* < 0.05 was deemed as statistically significant.

### Multivariate Machine Learning

Multivariate support vector classification (SVC) was carried out for the identification of EMCI from HC and least absolute shrinkage and selection operator (lasso) regression were used to predict the neurological measures by using information flow measures (both preferred information flow direction and index) in EMCI and HC. For SVC, preferred information flow measures with statistically significant differences between EMCI and HC were used. The leave-one-out cross validation (LOOCV) was adopted to evaluate the performance of SVC with classification accuracy, sensitivity, specificity, positive predictive value (PPV), and negative predictive value (NPV). For lasso regression, Pearson's correlation coefficients between predicted and actual neurological measures were used to evaluate the model performance.

The details of the image processing and analysis were described in Figure 1.

## Results

### Demographics and Clinical Variables

No difference was observed on age, sex, ADNI-EF, Aβ, Tau, and pTau between EMCI and HC. Lower ADNI-MEM score was observed in EMCI (0.5 ± 0.57) compared to HC (1.0 ± 0.55; *p* < 0.001).

### Information Flow Pattern in EMCI

As shown in Figure 2B, the preferred information flow direction from middle frontal gyrus to angular gyrus, from precentral sulcus to inferior occipital gyrus, from inferior occipital gyrus to Rolando sulcus, from inferior parietal gyrus to anterior insula gyrus, from precentral sulcus to superior temporal pole gyrus, from superior temporal pole gyrus to anterior insular gyrus, from lateral occipital gyrus to parieto-occipital sulcus, and from posterior cingulate gyrus to hippocampus decreased in EMCI compared to HC.

As shown in Figure 2B, the preferred information flow index decreased in the posterior insular gyrus, superior temporal pole gyrus, posterior cingulate gyrus, and cuneus gyrus and increased in the precentral sulcus, middle temporal gyrus, and thalamus in patients with EMCI compared to HC.

### Differentiation of EMCI From HC by Using Information Flow Measures

As shown in Table 2, a classification accuracy of 79.78%, the sensitivity of 85%, specificity of 75.51%, PPV of 73.91%, and NPV of 86.05% were achieved for differentiation of EMCI from HC by using the above preferred information flow directions (seven features), which showed a statistical difference between groups.

**Table 2:** Identification of EMCI by using information flow measures by SVC.

**Features**	**Accuracy**	**Sensitivity**	**Specificity**	**PPV**	**NPV**
Preferred information flow direction(FN = 7)	79.78%	85%	75.51%	73.91%	86.05%
Preferred information flow index(FN = 8)	76.40%	70%	81.63%	75.68%	76.92%

*EMCI, early mild cognitive impairment; SVM, support vector machine; PPV, positive predictive value; NPV, negative predictive value; FN, feature number; SVC, support vector classification*.

Classification accuracy of 76.40%, the sensitivity of 70%, specificity of 81.63%, PPV of 75.68%, and NPV of 76.92% were achieved for differentiation of EMCI from HC by using the above preferred information flow index (eight features), which showed a statistical difference between groups.

### Clinical Assessment of EMCI by Using Information Flow Pattern

**Figure 3:**
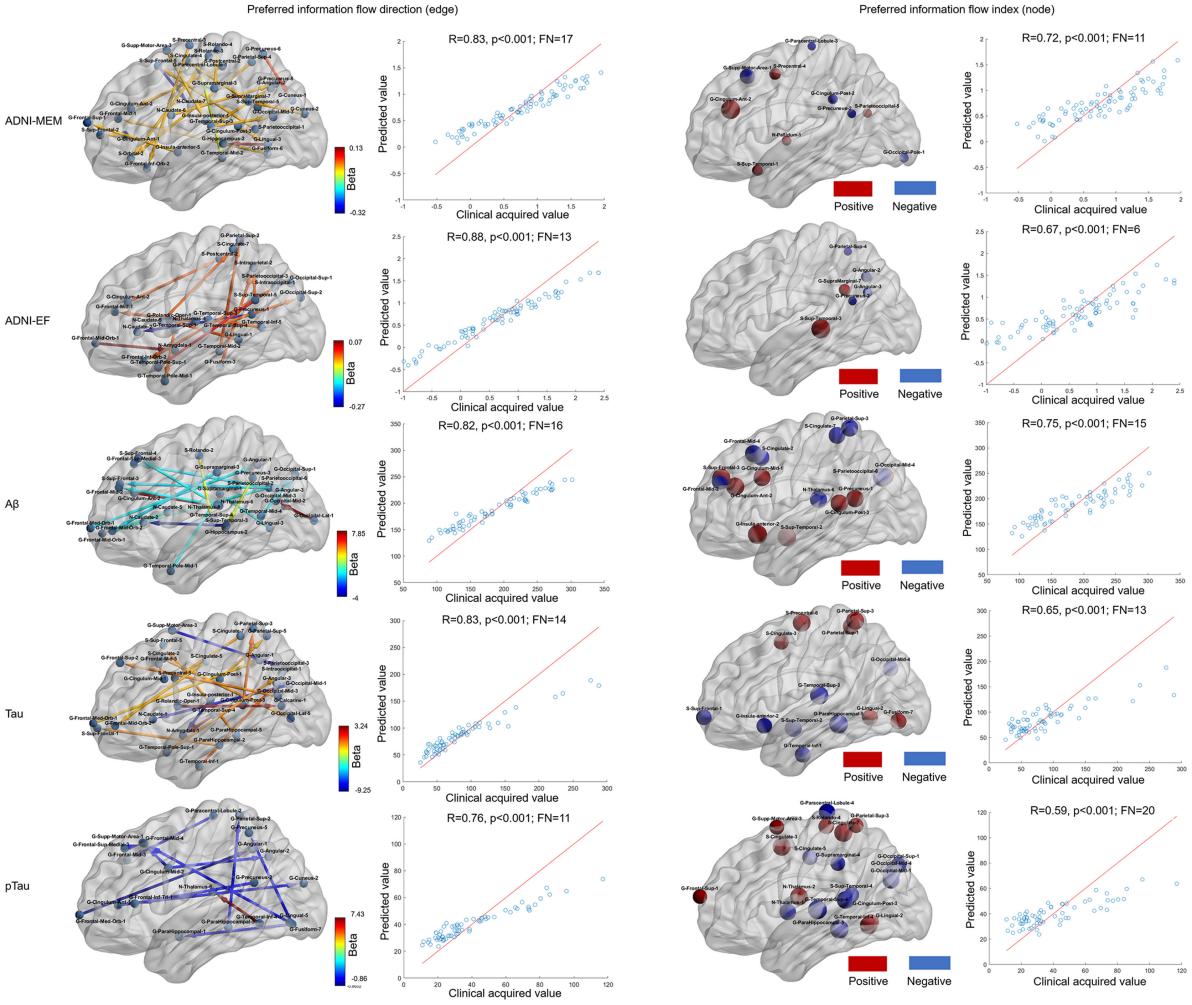
Neurological assessment by using information flow measures by lasso regression. The first and second columns presented the selected information flow directions and a good prediction ability for the ADNI-MEM, ADNI-EF, Aβ, tau, and pTau evaluation. The color of the directed edge indicated the value of lasso regression coefficient (beta); the third and fourth columns presented the selected information flow index and a relative decreased prediction ability for the ADNI-MEM, ADNI-EF, Aβ, tau, and pTau evaluation. The size of the node indicated the absolute value of the lasso regression coefficient and the color of the node indicated the positive (red) or negative (blue) lasso regression coefficient. Aβ, amyloid β; pTau, phosphorylated Tau; ADNI-MEM, Alzheimer's Disease Neuroimaging Initiative-composite assessment of memory; ADNI-EF, ADNI-executive function; FN, feature number; G, gyrus; S, sulcus.

As shown in Figure 3, when using preferred information flow directions (e.g., those between brain areas within frontal, temporal, and parietal lobe), the ADNI-MEM, ADNI-EF, level of Aβ, tau, and pTau could be well predicted with correlation coefficients of 0.83 (*p* < 0.001; feature number = 17), 0.88 (*p* < 0.001; feature number = 13), 0.82 (*p* < 0.001; feature number = 16), 0.83 (*p* < 0.001; feature number =1 4), and 0.76 (*p* < 0.001; feature number = 11), respectively. When using preferred information flow index (e.g., those of brain areas within frontal, temporal, and parietal lobe), the predictive ability decreased with lower correlation coefficients of 0.72 (*p* < 0.001; feature number = 10), 0.67 (*p* < 0.001; feature number = 6), 0.75 (*p* = 0.009; feature number = 15), 0.65 (*p* < 0.001; feature number = 13), and 0.59 (*p* = 0.01; feature number = 20), respectively.

## Discussion

In this study, the information flow pattern of EMCI was investigated by using preferred information flow direction and index between/within brain regions defined by NPMR-GCA and demonstrated their abilities for clinical differential diagnosis and neurological state prediction. Results showed disturbed preferred information flow directions involving default mode network (DMN), executive control network (ECN), somatomotor network (SMN), visual network (VN), and altered preferred information flow index in several brain areas (including the thalamus, posterior cingulate, and precentral gyrus) in EMCI compared to HC. Additionally, a good classification (accuracy of 80%) of EMCI and HC and good predictive abilities (*r* > 0.7) of the preferred information flow directions for the neurological state (cognitive measures and CSF biomarkers) were achieved.

The disturbed information flow pattern in EMCI was consistent with previous evidence that the alterations in patients with dementia predominately involved DMN, ECN, SMN, and VN (15, 19, 30, 31). The altered information flow pattern in cingulate, especially in posterior cingulate, was consistent with the functional deficit within DMN in patients with MCI and dementia (19, 32), which may account for the episodic memory problems in EMCI (33). In addition, the information flow in the temporal gyrus, angular gyrus, and insula further demonstrated the changes within DMN (19, 32, 34). The altered information flow in the frontal gyrus may associate with the mild cognitive decline in EMCI (18, 30). Information flow change in the precentral gyrus was observed in this study, implying a motor function deficit in patients with EMCI, which has been previously demonstrated (10). Occipital gyrus within VN showed disturbed information flow pattern in short- (e.g., between lateral occipital gyrus and parieto-occipital sulcus) and long-range (e.g., between precentral sulcus and middle occipital gyrus and between inferior occipital gyrus and Rolando sulcus) connectivity, which may account for the cognitive decline in EMCI (10, 19). Hippocampus, which was the most reported brain area in AD, also presented altered information flow directions with other brain regions, especially cingulate; this finding may account for the mild decline memory in patients with EMCI (34–36). The posterior cingulate, thalamus, precentral, insular, middle temporal gyrus, and temporal pole were acknowledged brain functional hubs in both healthy people and patients with neuropsychiatric disorders (15, 19), and the disturbances of the information flow in these brain hubs may account for the underlying pathological mechanism and clinical manifestation in patients with EMCI (1, 19, 37).

Based on the above findings, accurate differentiation of EMCI from HC was achieved. The classification accuracy (80%) was comparable to previous reports based on multimodal MRI (15, 34). Recently, deep learning based on fMRI demonstrated exciting performance for the early diagnosis of EMCI, achieving a classification accuracy of above 95%, which was superior to widely applied conventional machine learning methods (3, 4, 38). However, it was difficult to interpret the contributing features in deep learning models (27). Support vector machine (SVM) was a popular multivariate supervised data classification approach with performance being comparable or superior to other machine learning methods (e.g., k-nearest neighbor algorithm, Naive Bayes, decision trees, discriminant analysis), especially for small samples (39). The differentiation of EMCI from HC by using SVM further confirmed the clinical value of the information flow alterations in EMCI observed in this study.

The accurate prediction of neurological measures (reflecting neurological state) by using information flow patterns was achieved, which was rarely reported in previous MCI studies. These findings were of high importance, especially for those who were not available timely to clinical cognitive assessments and invasive CSF sampling, which can help physicians and clinicians for early screening of patients with EMCI. These findings also implied that the brain functional alterations of EMCI could provide objective radiological makers to assess the cognitive state and pathological changes in patients with EMCI, which was important for monitoring the disease progression, triaging for clinical trials, and evaluating the response to clinical treatments (2).

There are some limitations to this study. First, the sample size of included subjects was limited to Philips scanner and a specific protocol setting to avoid the potential influence of scanner and acquisition parameters, since this study was the first try to use NPMR-GCA to depict the underlying information flow in EMCI. In the future, larger samples by using different parameters on different MR scanners should be considered to validate the current findings. Second, the preferred information flow direction was a relative value between brain nodes, a value of 0.5 indicated no preferred information flow direction, which failed to characterize the information pattern of the brain nodes with simultaneous increased and decreased information outputting and receiving abilities. In addition, the information flow measures could not determine whether the increased preferred information flow direction was a result of underlying increased information outputting ability or decreased receiving ability. Third, LOOCV was adopted for the SVC model evaluation, overfitting might be presented especially for small samples, and further works with large samples and external testing datasets would be included to validate the current models. Lastly, this work aimed to investigate the intrinsic information flow revealed by rs-fMRI. As for the clinical diagnosis and neurological assessment, multimodal MRI including morphology, perfusion, and task-fMRI may improve the performance of the current diagnostic and predictive models, which would be conducted in the future with multimodal datasets from the multiple protocol settings and scanners.

## Conclusion

In this study, we defined preferred information flow direction and index by NPMR-GCA and observed their ability to help in the early diagnosis and neurological state assessment in EMCI, reflecting the underlying pathological process in patients with EMCI, which may help to guide the physician and clinician for early screening, monitoring disease progression, and providing objective biomarker for the clinical trials.

## Data Availability Statement

Publicly available datasets were analyzed in this study. This data can be found here: National Institute on Aging (NIA) Alzheimer's Disease Neuroimaging Initiative (ADNI), http://adni.loni.usc.edu/data-samples/access-data/.

## Ethics Statement

The studies involving human participants were reviewed and approved by Alzheimer's Disease Neuroimaging Initiative. The patients/participants provided their written informed consent to participate in this study.

## Author Contributions

HH contributed to the investigation, data processing, statistical analysis, and writing original draft. SD, CJ, Y-YW, and QL contributed to data processing and editing. Y-LW contributed to the investigation, conceptualization, data processing, review, and editing. All authors contributed to the article and approved the submitted version.

## Conflict of Interest

The authors declare that the research was conducted in the absence of any commercial or financial relationships that could be construed as a potential conflict of interest.

## Publisher's Note

All claims expressed in this article are solely those of the authors and do not necessarily represent those of their affiliated organizations, or those of the publisher, the editors and the reviewers. Any product that may be evaluated in this article, or claim that may be made by its manufacturer, is not guaranteed or endorsed by the publisher.
