# Supplementary material for: Information Flow Pattern in Early Mild Cognitive Impairment Patients
Source: Front Neurol. 2021 Nov 11;12:706631. doi: 10.3389/fneur.2021.706631 (PMC8631864; doi:10.3389/fneur.2021.706631)
Supplement: Supplementary file 1 [file Table_1.DOCX]

Code 1

function [Zxy Zyx Fx2y_L Fy2x_L Fx2y Fy2x]=NPMR_GCA(X,Y,order,alpha)

%X column vectors n*P with n points and P the number of the regression variates

%y column vectors n*1 for the prediction response

%X Y need subtract mean value

len=length(X);

Xlag=X(1:len-order,:);

Ylag=Y(1:len-order,:);

Xpre=X(1+order:len,:);

Ypre=Y(1+order:len,:);

for i=1:order

Xreg(:,i)=X(i:len-order+i-1,:);

Yreg(:,i)=Y(i:len-order+i-1,:);

end

XYreg=[Xreg Yreg];

XDist=pdist2(Xreg,Xreg);

YDist=pdist2(Yreg,Yreg);

ThetaXY=std(XYreg,0,1);

ThetaX=std(Xreg,0,1);

ThetaY=std(Yreg,0,1);

switch order

case 1

XDist=XDist./(ThetaX);YDist=YDist./(ThetaY);

WXY(:,1,:)=exp(-0.5*(XDist.^2));

WXY(:,2,:)=exp(-0.5*(YDist.^2));

Cum_WXY(:,:)=WXY(:,1,:).*WXY(:,2,:);

yWXY=Cum_WXY(:,:).*(repmat(Ypre,[1,length(Xpre)]));

xWXY=Cum_WXY(:,:).*(repmat(Xpre,[1,length(Xpre)]));

Cum_WX(:,:)=WXY(:,1,:);

xWX=Cum_WX(:,:).*(repmat(Xpre,[1,length(Xpre)]));

Cum_WY(:,:)=WXY(:,2,:);

yWY=Cum_WY(:,:).*(repmat(Ypre,[1,length(Xpre)]));

case 2

XDist1=pdist2(Xreg(:,1),Xreg(:,1));XDist2=pdist2(Xreg(:,2),Xreg(:,2));

YDist1=pdist2(Yreg(:,1),Yreg(:,1));YDist2=pdist2(Yreg(:,2),Yreg(:,2));

XDist1=XDist1./(ThetaX(1));YDist1=YDist1./(ThetaY(1));

XDist2=XDist2./(ThetaX(2));YDist2=YDist2./(ThetaY(2));

WXY(:,1,:)=exp(-0.5*(XDist1.^2));WXY(:,2,:)=exp(-0.5*(XDist2.^2));

WXY(:,3,:)=exp(-0.5*(YDist1.^2));WXY(:,4,:)=exp(-0.5*(YDist2.^2));

Cum_WXY(:,:)=WXY(:,1,:).*WXY(:,2,:).*WXY(:,3,:).*WXY(:,4,:);

yWXY=Cum_WXY(:,:).*(repmat(Ypre,[1,length(Xpre)]));

xWXY=Cum_WXY(:,:).*(repmat(Xpre,[1,length(Xpre)]));

Cum_WX(:,:)=WXY(:,1,:).*WXY(:,2,:);

xWX=Cum_WX(:,:).*(repmat(Xpre,[1,length(Xpre)]));

Cum_WY(:,:)=WXY(:,3,:).*WXY(:,4,:);

yWY=Cum_WY(:,:).*(repmat(Ypre,[1,length(Xpre)]));

case 3

XDist1=pdist2(Xreg(:,1),Xreg(:,1));XDist2=pdist2(Xreg(:,2),Xreg(:,2));XDist3=pdist2(Xreg(:,3),Xreg(:,3));

YDist1=pdist2(Yreg(:,1),Yreg(:,1));YDist2=pdist2(Yreg(:,2),Yreg(:,2));YDist3=pdist2(Yreg(:,3),Yreg(:,3));

XDist1=XDist1./(ThetaX(1));YDist1=YDist1./(ThetaY(1));

XDist2=XDist2./(ThetaX(2));YDist2=YDist2./(ThetaY(2));

XDist3=XDist3./(ThetaX(3));YDist3=YDist3./(ThetaY(3));

WXY(:,1,:)=exp(-0.5*(XDist1.^2));WXY(:,2,:)=exp(-0.5*(XDist2.^2));WXY(:,3,:)=exp(-0.5*(XDist3.^2));

WXY(:,4,:)=exp(-0.5*(YDist1.^2));WXY(:,5,:)=exp(-0.5*(YDist2.^2));WXY(:,6,:)=exp(-0.5*(YDist3.^2));

Cum_WXY(:,:)=WXY(:,1,:).*WXY(:,2,:).*WXY(:,3,:).*WXY(:,4,:).*WXY(:,5,:).*WXY(:,6,:);

yWXY=Cum_WXY(:,:).*(repmat(Ypre,[1,length(Xpre)]));

xWXY=Cum_WXY(:,:).*(repmat(Xpre,[1,length(Xpre)]));

Cum_WX(:,:)=WXY(:,1,:).*WXY(:,2,:).*WXY(:,3,:);

xWX=Cum_WX(:,:).*(repmat(Xpre,[1,length(Xpre)]));

Cum_WY(:,:)=WXY(:,4,:).*WXY(:,5,:).*WXY(:,6,:);

yWY=Cum_WY(:,:).*(repmat(Ypre,[1,length(Xpre)]));

end

for t=1:length(Xpre)

yY_est(t)=(sum(yWY([1:t-1,t+1:end],t)))./(sum(Cum_WY([1:t-1,t+1:end],t)));

xX_est(t)=(sum(xWX([1:t-1,t+1:end],t)))./(sum(Cum_WX([1:t-1,t+1:end],t)));

yXY_est(t)=(sum(yWXY([1:t-1,t+1:end],t)))./(sum(Cum_WXY([1:t-1,t+1:end],t)));

xXY_est(t)=(sum(xWXY([1:t-1,t+1:end],t)))./(sum(Cum_WXY([1:t-1,t+1:end],t)));

Rx2x(t)=Xpre(t)-xX_est(t);

Rx2xy(t)=Xpre(t)-xXY_est(t);

Ry2y(t)=Ypre(t)-yY_est(t);

Ry2xy(t)=Ypre(t)-yXY_est(t);

end

Zxy=sum((yY_est-yXY_est).^2);

Zyx=sum((xX_est-xXY_est).^2);

theta_x2x=sum((Rx2x-mean(Rx2x)).^2)./(len-order-1);

theta_x2xy=sum((Rx2xy-mean(Rx2xy)).^2)./(len-order-1);

theta_y2y=sum((Ry2y-mean(Ry2y)).^2)./(len-order-1);

theta_y2xy=sum((Ry2xy-mean(Ry2xy)).^2)./(len-order-1);

Fy2x_L=log(theta_x2x/theta_x2xy);

Fx2y_L=log(theta_y2y/theta_y2xy);

Fy2x=theta_x2x-theta_x2xy;

Fx2y=theta_y2y-theta_y2xy;

Code 2

clc;clear all

%permutation test for the significant analysis

%set data and save path

str2='Log';

save_path4=strcat('D:\Manuscripts\ADNI\Results\NC\NPMR_GCA');

%read data and related parameters;

cd(data_path);

file=dir('*.mat');

file_num=length(file);

tem=load(file(1).name);

data=tem.tem;

[m n]=size(data);

order1=1;%%%%%%%%%%%%%%%%%important

alpha=1;par=2;type='g';

clear tem data;

waitbar(0,'please waiting...');

for ni=1:file_num

%read data;

cd(data_path);

tem=load(file(ni).name);

data=tem.tem;

clear tem;

FX2Y=zeros(m,m);FX2YN=zeros(m,m);FX2YNN=zeros(m,m);

tic;

%calculation

for i=1:m-1

for j=i+1:m

X=data(i,:)';Y=data(j,:)';

X=X-mean(X);Y=Y-mean(Y);

[Zxy Zyx Fx2y_LNPMR Fy2x_LNPMR Fx2yNPMR Fy2xNPMR]=NPMR_GCA1(X,Y,order1,alpha);% Non-Parametric Multiplicative Regression, NPMR

Result_NPMR_GCA(i,j)=Fx2y_LNPMR; Result_NPMR_GCA(j,i)=Fy2x_LNPMR;

end

end

% %NPMR_GCA

cd(strcat(save_path4,'\',str2));

filename=strcat(file(ni).name);

save(filename,'Result_NPMR_GCA');

disp(strcat('Done_',file(ni).name));

waitbar(ni/file_num);

toc;

end

%

figure,imagesc(Result_NPMR_GCA);colorbar;

Code 3

clc;clear all;

datapath='D:\GCA\NPMR_GCA\Prefered_direction';

savepath='D:\GCA\NPMR_GCA\DTE\Prefered_direction';

cd(datapath);

filename=dir('*.mat');

num=length(filename);

Nlabel=90;

temp=load_nii('D:\template_3mm\AICHA.nii');

V=spm_vol('D:\template_3mm\AICHA.nii');

image=temp.img;

DTEALL=zeros(1,Nlabel);

DTEALL_image=zeros(size(image));

h=waitbar(0,'please waiting...');

figure,

for i=1:num

cd(datapath);

data=load(filename(i).name);

tem=data.FX2YNN_D;

tem(isnan(tem))=0;

outgoing=abs(sum((tem')));

incoming=abs(sum((tem)));

DTE=outgoing./(outgoing+incoming);

DTE1(i,:)=DTE;

DTEALL=DTE+DTEALL;

DTE_image=zeros(size(image));

for ii=1:Nlabel

DTE_image(image==ii)=DTE(ii);

end

DTEALL_image=DTE_image+DTEALL_image;

cd(savepath);

voxel_size=temp.original.hdr.dime.pixdim(2:4);

origin=[31 43 25];datatype = 64;

nii=make_nii(DTE_image,voxel_size,origin,datatype);

nii_name=strcat('DTE_',filename(i).name(1:end-4));

save_nii(nii,nii_name);

save(strcat(nii_name,'.mat'),'DTE');

waitbar(i/num);

end

DTEALL_image=DTEALL_image./num;

DTEALL=DTEALL./num;

cd(savepath);

nii=make_nii(DTEALL_image,voxel_size,origin,datatype);

nii_name='DTEALL_mean';

save_nii(nii,nii_name);

save(strcat(nii_name,'.mat'),'DTEALL');

save('DTE_mean.mat','DTE1');

save('DTE_mean.txt','DTE1','-ascii');
